# Random forest machine learning for maize yield and agronomic efficiency prediction in Ghana

**DOI:** 10.1016/j.heliyon.2024.e37065

**Published:** 2024-08-28

**Authors:** Eric Asamoah, Gerard B.M. Heuvelink, Ikram Chairi, Prem S. Bindraban, Vincent Logah

**Affiliations:** a Soil Geography and Landscape Group, Wageningen University & Research, PO Box 47, 6700, AA, Wageningen, the Netherlands; b Agricultural Innovation and Technology Transfer Center, Mohammed VI Polytechnic University, Lot 660, Hay Moulay Rachid, Benguerir, 43150, Morocco; c Council for Scientific and Industrial Research – Soil Research Institute, Kumasi, Ghana; d ISRIC – World Soil Information, PO Box 353, 6700, AJ, Wageningen, the Netherlands; e Modelling Simulation and Data Analysis, Mohammed VI Polytechnic University, Lot 660, Hay Moulay Rachid, Benguerir, 43150, Morocco; f International Fertilizer Development Center, Muscle Shoals, AL, 35662, USA; g Department of Crop and Soil Sciences, Kwame Nkrumah University of Science and Technology, Kumasi, Ghana

**Keywords:** Agronomic efficiency, Maize yield, Modelling, Random forest algorithm, Uncertainty assessment

## Abstract

Maize (*Zea mays*) is an important staple crop for food security in Sub-Saharan Africa. However, there is need to increase production to feed a growing population. In Ghana, this is mainly done by increasing acreage with adverse environmental consequences, rather than yield increment per unit area. Accurate prediction of maize yields and nutrient use efficiency in production is critical to making informed decisions toward economic and ecological sustainability. We trained the random forest machine learning algorithm to predict maize yield and agronomic efficiency in Ghana using soil, climate, environment, and management factors, including fertilizer application. We calibrated and evaluated the performance of the random forest machine learning algorithm using a 5 × 10-fold nested cross-validation approach. Data from 482 maize field trials consisting of 3136 georeferenced treatment plots conducted in Ghana from 1991 to 2020 were used to train the algorithm, identify important predictor variables, and quantify the uncertainties associated with the random forest predictions. The mean error, root mean squared error, model efficiency coefficient and 90 % prediction interval coverage probability were calculated. The results obtained on test data demonstrate good prediction performance for yield (MEC = 0.81) and moderate performance for agronomic efficiency (MEC = 0.63, 0.55 and 0.54 for AE-N, AE-P and AE-K, respectively). We found that climatic variables were less important predictors than soil variables for yield prediction, but temperature was of key importance to yield prediction and rainfall to agronomic efficiency. The developed random forest models provided a better understanding of the drivers of maize yield and agronomic efficiency in a tropical climate and an insight towards improving fertilizer recommendations for sustainable maize production and food security in Sub-Saharan Africa.

## Highlights

• Random forest modelling of maize yield in Ghana was successful and explained 81 % of the variance.
• Random forest modelling of agronomic efficiency was less accurate than for yield and explained between 53 and 63 % of the variance.
• Soil variables were more important than climate and other environmental variables in predicting yield.
• The random forest model can guide the development of fertilizer recommendations for sustainable maize production.

## 1 Introduction

In the era of increasing global population, ensuring food security has become a major challenge for scientists, governments, and non-governmental organizations [1]. It is projected that the world population will reach approximately 8.5 billion by 2030 and 9.7 billion by 2050 [2]. More than half of this increase will come from Sub-Saharan Africa (SSA), which poses a threat to food security in the region unless critical measures are taken to produce enough food for the growing population [3]. The consumption of cereals in SSA is increasing faster than its production, resulting in an over-reliance on imports [3]. This situation is exacerbated by the impact of climate change, which poses a significant threat to food security in SSA [4].

Maize is a crucial staple crop grown in all agro-ecological zones of Ghana and is the most consumed crop in the country [5]. Maize makes up over 50 % of the country's cereals production, providing an essential feed source for the livestock and poultry industries [5]. It is cultivated on approximately 25 % of Ghana's total arable land [6]. The increase in maize production has been primarily driven by land expansion rather than yield improvement, with negative impact on biodiversity and soil organic carbon content [7]. [8] attribute low maize yields in Ghana to factors such as drought, pest and disease infestations, poor soil fertility, inadequate use of fertilizers, and insufficient farmer adoption of good management practices. Understanding the relationships between these factors and yield can significantly inform farmers and other stakeholders on the drivers of maize yields, enhancing relevant decisions to making Ghana self-sufficient in maize production [9,10].

Agronomic efficiency (AE) is a measure of the yield increase achieved per unit nitrogen (N), phosphorus (P) and potassium (K) applied. Conceptually, crop yield is made up of two elements [11]. The first element is the yield produced by the soil's natural supply of nutrients, while the second is the yield increase resulting from fertilizer application. Agronomic efficiencies of N, P and K are affected by climate, soil, and management practices, which can vary among smallholder farms [12,13]. Adequate crop information and understanding the relationships between yield, applied nutrients, soil and climatic conditions, environmental factors, and management practices that influence AE are key for sustainable agriculture [14]. Identifying these drivers can assist decision-makers in determining the ideal nutrient combination and management for maximizing yields and improving AE.

Machine learning-based models have been recognized for their high potential for crop modelling in recent scientific literature. For example [15], used a support vector machine model to predict rice development stage and yield using meteorological data [16]. evaluated various machine learning models, including decision trees (DT), random forest (RF), support vector machine (SVM), Bayesian networks (BN), and artificial neural networks (ANN), to predict crop yields based on climatic and soil data [17]. successfully used the RF algorithm to predict seasonal variations in sugarcane yield using simulated biomass from the Agricultural Production Systems sIMulator (APSIM), seasonal climatic indices, and weather data in Northeastern Australia [18]. evaluated the RF algorithm for predicting wheat yield in southeast Australia using normalized difference vegetation index (NDVI) data derived from high-resolution satellite imagery and weather data. Among the various ML models, RF has proven to perform equally well as other machine learning models in predicting yields of maize, wheat, mango, potato, sugarcane, and rice using environmental and climatic variables [[19], [20], [21], [22], [23], [24], [25]]. The RF algorithm is computationally attractive and stands out for its ability to explore non-linear relationships between predictor and response variables using an ensemble approach [17]. However, to the best of our knowledge, no study has used the RF algorithm to predict both yield and AE for maize production in SSA.

**Fig. 1:**
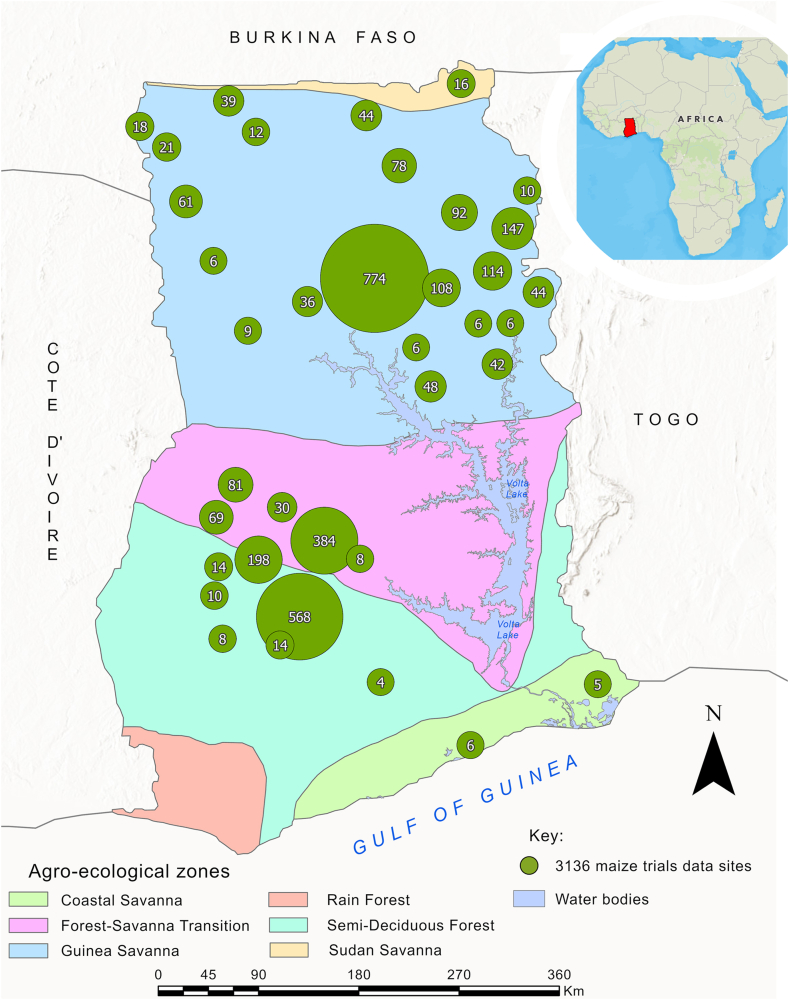
Map showing locations of maize-treatment plots (n = 3136) from 482 fertilizer experimental trials across five agro-ecological zones of Ghana.

Uncertainty assessments are crucial in model predictions to inform decision making [26], yet previous studies have not thoroughly considered uncertainties in yield predictions. Quantifying prediction uncertainties with the RF algorithm can be achieved with the quantile regression forest (QRF) approach, which estimates the conditional probability distribution of the response variable [27]. The QRF provides estimates of prediction intervals which gives a measure of the uncertainty associated with each prediction and also provides insights into how the uncertainty in predictions varies across different regions of the feature space [28]. Much work has been done on using the RF algorithm for yield prediction [29]. However, there is limited information in the literature regarding the AE of N, P, and K predictions, as well as estimating the uncertainties in the models’ predictions. In this study, we took advantage of the availability of comprehensive datasets from across the country (Fig. 1) to develop a predictive model for maize yield and agronomic efficiency for Ghana.

The objectives of this study were to: (i) collect and harmonize data on maize yield, fertilizer application, and environmental variables in Ghana; (ii) calibrate a RF algorithm using hyperparameter optimization and assess the performance of the calibrated RF algorithm for yield and AE prediction through cross-validation; (iii) quantify and evaluate the predictive uncertainty of the RF algorithm for yield and AE prediction using quantile regression forest; and (iv) determine and interpret the relative importance of the RF predictor variables for yield and AE prediction.

## 2 Materials and methods

### 2.1 Study area

Ghana is located in West Africa between latitude 4° 11′ N and 11° 11′ N and longitude 3° 11′ W and 1° 11′ E. It shares borders with Togo in the east, Cote d’Ivoire in the west, and with Burkina Faso in the north. In the south, Ghana is bordered by the Gulf of Guinea. The total land area is 238,533 km^2^, with a population of a little over 30 million, as revealed by the 2021 population census [30]. The study area included all agro-ecological zones of Ghana, namely the Guinea Savanna (GS), Sudan Savanna (SS), Forest-Savanna Transition (FST), Semi-Deciduous Forest (SDF) and the Coastal Savanna (CS) zones, except the Rain Forest (RF) (Fig. 1). The SS and GS have one major annual planting season, starting in May and ending in October. FST, SDF, RF and CS have two planting seasons, a major season from April to July, and a minor season from September to November. Table 1 shows general characteristics of each agro-ecological zone.

**Table 1:** General characteristics of the agro-ecological zones in Ghana.

Agro-ecological zone	Rainfall range (mm year^1^)	Mean temperature range (°C year^−1^)	Length of growing season (days)	Major land use systems	Major soil type (WRB Reference Soil Groups)
Sudan Savanna	900–1100	26–32	MJ: 180–200	Annual food crops, cash crops, livestock	Lixisol, Plinthosol, Luvisol
Guinea Savanna	1000–1200	26–32	MJ: 190–230	Annual food crops, cash crops, livestock	Lixisol, Planosol, Plinthosol
Forest-Savanna Transition	1100–1400	24–28	MJ: 130–200 MN: 70	Annual food crops, cash crops	Lixisol, Plinthosol
Semi-Deciduous Forest	1200–1500	24–28	MJ: 130–160 MN: 80	Annual food crops, forest, plantations	Acrisol, Lixisol, Nitisol
Coastal Savanna	800–1000	26–32	MJ: 100–110 MN: 50	Annual food crops	Vertisol, Luvisol, Cambisol
Rain Forest	1700–2300	24–28	MJ: 90–120 MN: 40	Forest, plantations	Ferralsol, Acrisol, Gleysol

MJ: Major season, MN: Minor season. Source: Modified after [6], WRB – World Reference Base for Soil Resources [31].

### 2.2 Datasets and data sources

#### 2.2.1 Maize trials data and predictor variables

Data used to model and predict maize yield and AE were compiled from three sources: the International Fertilizer Development Center (IFDC) database [32], National Research Institutes and Universities (NRI&U) in Ghana, and the IFDC – Fertilizer Research and Responsible Implementation (FERARI) project (https://ifdc.org/projects/fertilizer-research-and-responsible-implementation-ferari/). The data from the IFDC database consisted of 263 maize field trials data retrieved from peer-reviewed publications from scientific databases including Google Scholar, Web of Science, Scopus, African Journals Online and the Food and Agriculture Organization of the United Nations. The data from the NRI&U database were derived from 86 field trials retrieved from unpublished Master's and Doctoral theses from three public universities in Ghana, namely Kwame Nkrumah University of Science and Technology, University of Ghana, and University for Development Studies. Finally, the data from the IFDC-FERARI project consisted of 133 maize field trials conducted in 2020. We harmonized the maize field trial datasets from these three data sources into one database. The moisture content at which grain yield was reported ranged from 13 to 15 % in the compiled harmonized database. We preprocessed the data to conform to the same standard units for variables and removed redundant information from the combined database. This resulted in 3136 unique georeferenced plot data points from 1991 to 2020 (Table 2 and Fig. 1).

**Table 2:** Sources for fertilizer and maize yield data compilation.

Data source	Number of field trials	Number of treatment plots	Reference
IFDC	263	919	Compiled from published journal articles [32]
NRI&U	86	1017	Compiled from national research institutes (CSIR-SRI, CSIR SARI) and universities (KNUST, UG, UDS)
IFDC – FERARI Project	133	1200	Compiled from FERARI project 2020 field trials
Total	482	3136	

CSIR-SRI: Council for Scientific and Industrial Research – Soil Research Institute, CSIR SARI: Council for Scientific and Industrial Research – Savanna Agriculture Research Institute, KNUST: Kwame Nkrumah University of Science and Technology, UG: University of Ghana, UDS: University for Development Studies.

Predictor variables identified to influence yield and AE were climatic variables, soil variables, crop genotype, environmental variables, management practices, and fertilizer application data. Forty predictor variables were prepared for the modelling. A summary of predictor variables is presented in Table 3, while Supplementary Information (SI) Tables SI 1-5 provide general research trial information and a detailed description of the predictor variables. Data collection strategies for three of the predictor variable groups are explained in Sections 2.2.2, 2.2.3.

**Table 3:** Predictor variables used in the RF algorithm prediction.

Variable groups (number of predictor variables)	Variables
Climate (6)	Rainfall (annual and total for planting season), temperature at planting season (minimum and maximum), mean relative humidity at planting season, mean evapotranspiration at planting season
Soil (0–30 cm) (21)	pH, organic carbon, total nitrogen, cation exchange capacity, available phosphorus, exchangeable bases (calcium, potassium, magnesium and sodium), sand, silt, clay, bulk density, coarse fragment content, electrical conductivity, zinc, iron, total exchangeable bases, base saturation, root zone water holding capacity, soil type
Crop (1)	Genotype
Environmental (3)	Slope, NDVI, Agro-ecological zone
Management practices (3)	Application of any organic amendment (e.g. poultry manure, cattle manure), management type, mode of fertilizer application
aFertilizer application (6)	Nitrogen, phosphorus, potassium, sulphur, zinc, iron

a Only considered in predicting yield and not in predicting agronomic efficiencies (see Supplementary Information for a complete list of predictor variables).

#### 2.2.2 Climatic data

Climatic data (Table 4) for each experimental trial were obtained for the planting season of the trial, and values were aggregated over time to correspond to the time period of each trial. Climate station data closest to the experimental trial were obtained from the Ghana Meteorological Service (GMet) for experiments without climate information. Data from 1991 to 2020 were obtained from the GMet archive.

**Table 4:** Climatic information for major and minor planting seasons for the agro-ecological zones in Ghana.

Planting Season	Agro-ecological zone	T min (°C)	T max (°C)	RH-mean (%)	Et (mm)	R (mm)
Major	Sudan Savanna	22.9	32.7	70.3	154.7	897.5
	Guinea Savanna	22.6	31.5	76.1	149.9	938.9
	Forest-Savanna Transition	21.8	31.1	74.3	135.5	703.7
	Semi-Deciduous Forest	21.9	30.8	78.8	137.2	809.6
	Coastal Savanna	23.8	30.6	79.0	152.0	572.8
Minor	Forest-Savanna Transition	20.6	30.0	79.5	113.0	430.1
	Semi-Deciduous Forest	21.3	30.1	75.6	124.3	423.4
	Coastal Savanna	22.8	29.8	79.7	147.1	184.9

T min: minimum temperature, T max: maximum temperature, RH-mean: mean relative humidity, Et: mean evapotranspiration, R: rainfall.

#### 2.2.3 Soil data and other environmental variables

Soil fertility information of the tilled layer (0–30 cm) was extracted from the Ghana Soil Information Service (GhaSIS) hosted by CSIR-SRI (www.csirsoilinfo.org). The soil type (Reference Soil Group) [31] for each site was identified using the soil map of Ghana (Figure SI 4). Extracted soil fertility information from the existing GhaSIS database was used to fill gaps for sites where such information was missing. Other environmental variables used in the modelling were the slope [33] and the NDVI [34].

### 2.3 Agronomic efficiency (AE)

The nutrient use efficiency indicator modelled in this study was AE. AE is defined as the unit increase in yield per unit of nutrient applied [35] as in Eq. (1):

(1) $$\text{AE}=\frac{Y_{t}-Y_{c}}{F}$$

where $Y_{t}$ is the grain yield (kg ha^−1^) from the treatment plot, $Y_{c}$ is the grain yield (kg ha^−1^) from the control plot, and F refers to the fertilizer input (kg ha^−1^). We computed the AE of N, P, and K, and thus, yielding three agronomic efficiencies (AE–N, AE–P, and AE–K). The total number of observations used for calculating AE–N, AE–P, and AE–K were 2145, 1897 and 1799, respectively.

### 2.4 Random forest modelling

**Fig. 2:**
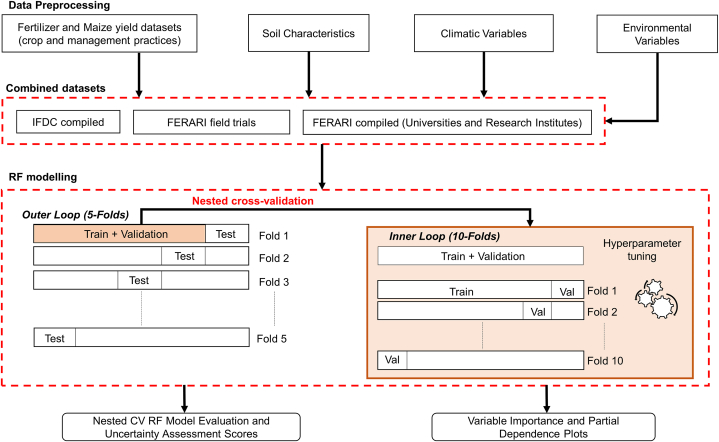
Flow diagram for the RF modelling.

RF is an ensemble-tree technique developed by Breiman [36]. It predicts the dependent variable by averaging decision tree predictions. Each tree is trained using a bootstrap sample from the training set and using a randomly sampled subset of the predictor variables. Each branch node in a tree represents a choice between two alternatives, and each leaf node represents a decision. The RF can identify linear and non-linear relationships between variables for classification and regression purposes. We used RF for regression to predict maize yield and AE from the predictor variables. All predictor variables (Table 3) were considered in predicting yield, but for AE, fertilizer application rates were excluded. Fertilizer application was not used as a predictor variable for predicting the agronomic efficiencies as this is used in the definition of the AE (see Eq. (1)). Predictor variables with zero and near-zero variance were not used for the RF predictions. Fig. 2 provides an overview of the RF modelling process used in this study.

#### 2.4.1 Hyperparameter tuning and model evaluation

Hyperparameter tuning aims at finding the optimal set of hyperparameter values that maximize the model's predictive performance [37]. We conducted a full cartesian grid search for the hyperparameters (Table 5) using a nested cross-validation [38]. The number of trees in the forest was not optimized but set to a sufficiently large value (1000 trees) to ensure that it did not decrease the predictive performance [39].

**Table 5:** Overview of the RF hyperparameters and their values included in optimization.

Hyperparameter	Description	Evaluated values
*Mtry*	Number of randomly drawn candidate variables in each split for growing a tree	$\sqrt{V}$, 25 %, 33.3 % and 40 % of V
Node size (*minimum.node.size*)	Minimum number of observations in a terminal node	1, 3 and 5
Replace	Sampling approach	TRUE (sample with replacement) and FALSE (sample without replacement)
Sample.fraction	Fraction of observations in the calibration dataset to sample in each tree	0.50, 0.63 and 0.80

V: number of predictor variables.

The performance of the models was evaluated using a 5 × 10-fold nested cross-validation approach. Nested cross-validation is a technique for performing hyperparameter tuning and model evaluation on separate datasets. It ensures that the test data are not in any way used in the modelling and hyperparameter estimation. In this way, unbiased estimates of the model performance metrics can be obtained [40]. The steps followed for the 5 × 10 nested cross-validation implementation are outlined as follows:

i. The data were repeatedly split into an outer and inner loop. The outer loop was used for evaluating the model, while the inner loop was used for hyperparameter tuning. In the outer loop, the data were split into 5-folds and each fold was once held out as a test dataset, while the remaining 4-folds were merged.
ii. Each of the 4 merged outer folds was split into 10 inner folds for training and hyperparameter estimation. We trained the model on a merge of 9 inner folds and evaluated the performance for each hyperparameter combination on the remaining inner fold. The process was repeated 10 times so that each inner fold was used once. In other words, for each combination of hyperparameters, we performed 10-fold cross-validation on the inner folds and recorded the average performance across all 10-folds.
iii. The hyperparameters of the RF algorithm with the highest frequency based on performance in the 10-fold inner cross-validation were selected.
iv. The selected hyperparameters were used to calibrate the model on 4 outer folds and tested on the remaining outer fold, and the predictions recorded. This was done 5 times, so that all folds were used for testing once.

#### 2.4.2 Model evaluation

We used the mean error (ME), the root mean square error (RMSE), and model efficiency coefficient (MEC) as evaluation metrics to assess the performance of the RF algorithm for yield and AE prediction based on the test data. The ME measures the systematic difference between the predicted and measured values as shown in Eq. (2). The RMSE measures the average magnitude of the errors in the predictions as shown in Eq. (3). The MEC measures how well a model predicts the dependent variable compared to just taking the average of the test data, as shown in Eq. (4). A MEC of 1 indicates perfect model performance, while a value of 0 indicates that the model has poor performance and does not improve on taking the average. The performance of the models was also visualized using scatter density plots of predicted against measured values.

(2) $$\text{ME}=\frac{1}{n}\;\sum_{i=1}^{n}\left(yᵢ-\;ŷᵢ\right)\;$$

(3) $$\text{RMSE}=\sqrt{\frac{1}{n}\;\sum_{i=1}^{n}\left(yᵢ-\;ŷᵢ\right)}²$$

(4) $$\text{MEC}=1-\;\frac{\sum_{i=1}^{n}\left(yᵢ-\;ŷᵢ\right)²}{\sum_{i=1}^{n}\left(yᵢ-\;ȳ\right)²}$$

where n is the number of trial plots, $y_{i}$ and ${ŷ}_{i}$ are the measured and predicted dependent variable at the i-th trial plot, respectively, and ȳ is the mean of the measurements.

#### 2.4.3 Uncertainty quantification

To quantify the uncertainty of the RF algorithm predictions for yield and AE, we used QRF [27]. QRF generates the quantiles of the conditional probability distribution of the variable of interest. From these quantiles, we computed prediction intervals (PI) to measure the uncertainty of the predictions. The 90 % prediction interval (PI90) was computed using the 0.05 and 0.95 quantiles of the conditional distribution. The width of the PI90 was then calculated as shown in Eq. (5).

(5) $$\text{PIW}=q_{0.95}-q_{0.05}$$

The PIW represents the uncertainty associated with each model prediction. To evaluate these uncertainty estimates, PIs were defined for various prediction levels, and the Prediction Interval Coverage Probability (PICP) was calculated for each level. The PICP measures the proportion of true measurements that fall within a PI [26] and it assesses whether the PI accurately represents the prediction uncertainty. For instance, approximately 90 % of the test data are expected to fall within the PI90, that is the 90 % prediction interval, indicating that ideally the PICP of the PI90 should be 0.90. Therefore, a substantially smaller or bigger PICP than the nominal value indicates that the model is not providing reliable uncertainty estimates. Multiple PICPs were calculated for different PI levels to evaluate the reliability of the entire predictive distribution. Accuracy plots were utilized to provide a graphical assessment of the model's performance for all PI levels [41]. Ideally, the PICP line shown in an accuracy plot should be close to the 1:1 line [42]. A PICP line below the 1:1 line indicates an underestimation of prediction uncertainty, a PICP line above the 1:1 line suggests an overestimation of prediction uncertainty [43].

#### 2.4.4 Variable importance and partial dependence plots

In addition to making predictions, RF also provides information about variable importance, which is useful for model interpretation. Identifying the most important predictor variables gives insight into the underlying mechanisms, although one must be careful when interpreting these because they do not necessarily reflect causal relationships. We implemented the permutation-based approach to determine the variable importance of each predictor variable [44].

We also used partial dependence plots (PDPs) [45] to gain insight into the impact of the topmost important variables on yield and AE as determined by the RF algorithm. Partial dependence plots visually depict the functional relationship between a predictor variable of interest and the dependent variable (i.e., yield and AE), while controlling for the effect of other predictor variables [45]. The partial dependence is estimated by marginalizing the predicted targets based on the distribution of the other predictor variables. Therefore, the PDP illustrates how the dependent variable changes with changes in the selected predictor variable.

### 2.5 Software implementation

Data preprocessing, exploratory data analysis and modelling were done using the R software for statistical computing (version 4.2.3) [46] integrated with RStudio. Data cleaning, handling and structuring were performed using the tidyverse and dplyr packages [47]. Data exploration was done using the dlookr package [48]. Handling of spatial and raster datasets was performed using the terra package [49]. Graphics and visuals were created with the base R package and ggplot2 [47]. The caret [50] and ranger [51] packages were used to build the RF algorithm. We used the ranger package with ‘quantreg’ to apply the quantile regression forest approach to quantify prediction uncertainties. We use the pdp package in R to calculate the PDPs for our analysis.

## 3 Results

### 3.1 Descriptive statistics of the datasets: dependent and predictor variables

**Fig. 3:**
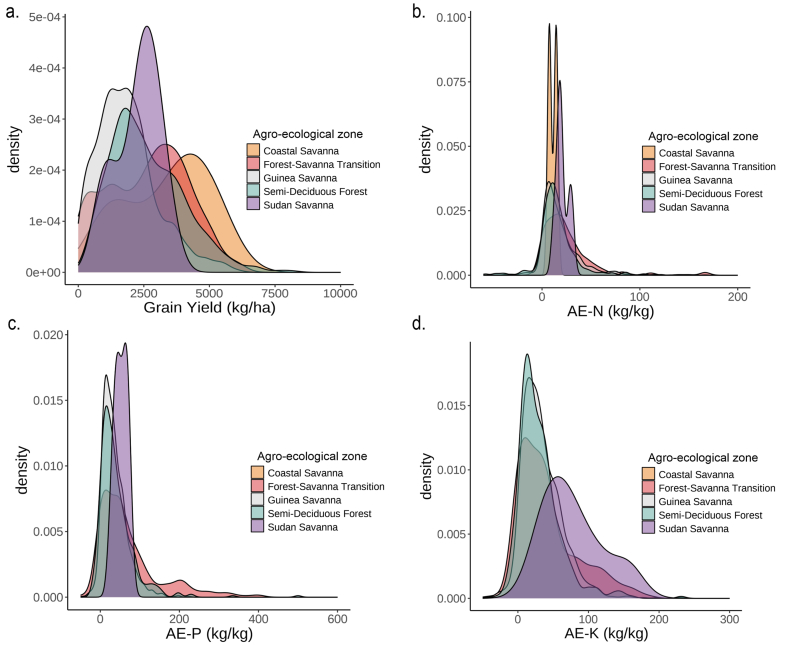
Density plots of a) maize yield, b) AE-N, c) AE-P, and d) AE-K across the agro-ecological zones of Ghana.

The search for data on maize trials conducted across Ghana's agro-ecological zones yielded data from 3136 plots. As explained in Section 2.2.1, the compiled data from research institutes and universities contained some missing data, mostly for soil properties, which were filled with information from soil property maps for Ghana developed by CSIR-SRI. The gap filling percentages for soil properties, namely phosphorus, exchangeable potassium, calcium, magnesium; pH, soil organic carbon, and total nitrogen, were 25 %, 21 %, 30 %, 31 %, 17 %, 20 %, and 17 %, respectively. Table 6 shows that the number of measurements for the AE variables were lower than for yield, since these were derived from comparing the yield at a nutrient treatment plot with that of a control plot, as explained in Section 2.3. The median grain yield across all experimental plots was 2000 kg ha^−1^ (Table 6), with yield ranging from 11 kg ha^−1^ to 8230 kg ha^−1^ (Table 6, Fig. 3a). Summary statistics and boxplots of the yield and agronomic efficiencies for different values of the predictor variables are presented in Table 6 and Tables SI 6–12 and Figures SI 1–3, respectively.

**Table 6:** Summary statistics of yield, AE and continuous-numerical predictor variables included in the RF yield and AE modelling.

Class		Variables	Unit	n	Min	Q1	Mean	Median	Q3	Max	SD	IQR	Skewness
Dependent variables		Grain yield	kg ha^−1^	3136	11	1238	2222	2000	3050	8230	1337	1811	0.7
		AE–N	kg kg^−1^	2145	−66.6	6.3	18.8	14.1	25.0	222.2	22.5	18.8	2.8
		AE–P	kg kg^−1^	1897	−57.6	13.5	43.0	31.6	56.3	606.7	50.1	42.8	3.4
		AE–K	kg kg^−1^	1799	−57.6	12.5	34.2	27.9	48.9	335.0	32.4	36.4	1.8
Predictor variables	Climate	T min PS	°C	3136	18.0	21.8	22.3	22.3	22.7	31.9	0.9	0.9	1.9
		T max PS	°C	3136	27.0	30.0	30.9	31.0	31.0	40.0	1.3	1.0	0.8
		RH mean	%	3136	61.9	78.8	78.8	78.8	78.8	90.0	3.5	0.0	−0.7
		RA PS	mm	3136	441	593	707	724	825	940	142	232	−0.3
		AR	mm	3136	810	1276	1276	1276	1276	1723	104	0	−0.1
		Av ET	mm	3136	103.9	136.2	136.2	136.2	136.2	156.1	5.1	0.0	−2.7
	Soil	pH	–	3136	4.1	5.7	5.9	6.0	6.1	7.3	0.4	0.4	−0.6
		SOC	%	3136	0.16	0.55	0.84	0.68	0.82	4.30	0.63	0.27	3.4
		Total N	%	3136	0.0	0.06	0.07	0.07	0.07	0.30	0.03	0.02	2.2
		CEC	cmol_+_ kg^−1^	3136	0.08	5.39	7.44	6.29	7.45	82.90	7.79	2.06	8.7
		Av P	mg kg^−1^	3136	0.0	3.7	24.5	18.1	23.9	379.5	57.2	20.2	5.5
		Ex K	cmol_+_ kg^−1^	3136	0.01	0.12	1.79	0.22	1.79	37.0	5.98	1.67	5.5
		Ex Ca	cmol_+_ kg^−1^	3136	0.09	0.14	1.51	1.52	1.52	11.71	1.66	1.38	2.2
		Ex Mg	cmol_+_ kg^−1^	3136	0.02	0.06	0.49	0.49	0.49	3.40	0.52	0.43	1.8
		Sand	%	3136	40.0	58.8	64.8	64.8	70.5	93.0	8.4	11.7	0.0
		Clay	%	3136	4.0	16.2	22.5	22.4	29.8	52.0	9.1	13.6	0.2
		Silt	%	3136	2.2	14.1	21.9	23.2	27.1	48.1	8.8	13.0	0.0
		BD	g cm^−3^	3136	1.12	1.21	1.34	1.34	1.47	1.67	0.13	0.26	0.1
		TEB	cmol_+_ kg^−1^	3136	0.18	0.40	0.41	0.41	0.41	0.81	0.10	0.0	2.1
		RZWHC	cm	3136	9.0	10.4	10.4	10.4	10.4	13.0	0.5	0.0	0.4
		BS	%	3136	24.1	49.6	49.5	49.6	49.6	82.3	10.5	0.0	0.1
		CsFrg	%	3136	13.0	38.2	44.1	45.2	49.9	59.6	9.0	11.6	−0.8
		Ex Na	cmol_+_ kg^−1^	3136	0.11	0.18	0.26	0.22	0.26	1.47	0.16	0.08	4.5
		EC	mS m^−1^	3136	0.05	0.14	1.21	0.17	1.21	34.22	3.36	1.07	6.7
		Zn	mg kg^−1^	3136	0.3	1.5	1.8	1.8	1.8	8.5	1.3	0.4	3.3
		Fe	mg kg^−1^	3136	1.4	33.7	33.7	33.7	33.7	115.9	14.4	0.0	1.3
	Fertilizer nutrient	Zn	kg ha^−1^	3136	0	0	0	0	0	10	1	0	2.3
		S	kg ha^−1^	3136	0	0	2	0	0	15	5	0	2.0
		Fe	kg ha^−1^	3136	0	0	0	0	0	5	1	0	3.6
		N	kg ha^−1^	3136	0	18	67	60	120	281	51	102	0.2
		P_2_O_5_	kg ha^−1^	3136	0	0	24	20	40	120	22	40	0.6
		K_2_O	kg ha^−1^	3136	0	0	24	25	40	120	23	40	0.5
	Environment	Slope	%	3136	0.0	0.6	1.3	0.9	1.7	6.0	1.2	1.1	2.1
		NDVI	–	3136	0.2	0.4	0.4	0.4	0.4	0.6	0.1	0.0	−0.8

n: Sample size, Min: minimum, Q1: first quartile, Q3: third quartile, Max: maximum, SD: Standard Deviation, IQR: inter-quartile range, AE-N: Agronomic efficiency of nitrogen, AE-P: Agronomic efficiency of phosphorus, AE-K: Agronomic efficiency of potassium, T min PS: minimum temperature in planting season, T max PS: maximum temperature in planting season, RH mean: mean relative humidity, RA PS: total rainfall amount in planting season, AR: total annual rainfall, Av ET: average evapotranspiration, SOC: soil organic carbon, Total N: soil total nitrogen, CEC: cation exchange capacity, Av P: soil available phosphorus, Ex K: exchangeable potassium, Ex Ca: exchangeable calcium, Ex Mg: exchangeable magnesium, BD: bulk density, TEB: total exchangeable bases, RZWHC: root zone water holding capacity, BS: base saturation, CsFrg: coarse fragment, Ex Na: exchangeable sodium, EC: electrical conductivity, Zn: Zinc, Fe: Iron, S: Sulphur, N: nitrogen, NDVI: normalized difference vegetation index. See Supplementary Information for explanation of the variables.

### 3.2 RF modelling

#### 3.2.1 Best RF tuning hyperparameters for yield and agronomic efficiency

A 10-fold cross-validation was used to optimize the hyperparameters of the RF algorithm for yield and agronomic efficiency. A full Cartesian grid search was employed to search for the best combination of hyperparameters. The optimized parameters are presented in Table 7.

**Table 7:** Optimized hyperparameter combination selected by maximum occurrence in the 5 × 10-fold nested cross-validation for yield and agronomic efficiency RF modelling.

RF Algorithms		Yield	AE–N	AE–P	AE–K
Hyperparameters	mtry	6	5	5	5
	minimum node size	3	5	5	5
	replace	FALSE	FALSE	FALSE	FALSE
	sample.fraction	0.8	0.8	0.8	0.8

#### 3.2.2 Predictive performance

**Fig. 4:**
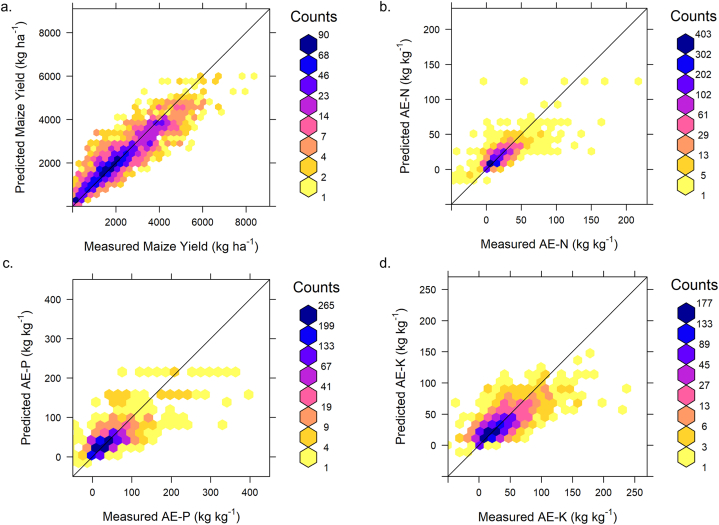
Scatter density plots (predicted vs measured) of RF algorithm for a) Maize yield, b) AE–N, c) AE–P, d. AE–K.

The results of the four RF models (yield, AE–N, AE–P, and AE–K) showed varying performance on the test data (Table 8 and Fig. 4). The yield model showed that systematic errors in the yield predictions were small as the ME was 0.185 kg ha^−1^ and negligibly small compared to the RMSE. The mean errors for the agronomic efficiency of N, P and K models were also small (i.e., nearly zero), showing unbiased predictions. The RMSE for the yield model was 582.2 kg ha^−1^, which is substantial but considerably smaller than the yield standard deviation of 1337 kg ha^−1^ (Table 6). The RMSEs for the agronomic efficiency models ranged from 13.7 to 33.5, with AE-N having the smallest RMSE and AE-P, the largest RMSE. The MECs for all AE models ranged between 0.54 and 0.63, while the yield model had the highest MEC with the model explaining 81 % of the variance.

**Table 8:** RF algorithm performance for maize yield and agronomic efficiency predictions.

RF Algorithms		Yield (kg ha^−1^)	AE–N (kg kg^−1^)	AE–P (kg kg^−1^)	AE–K (kg kg^−1^)
Model performance metric	ME	0.185	0.001	−0.017	−0.005
	RMSE	582.2	13.7	33.5	22.0
	MEC	0.810	0.630	0.554	0.536
Uncertainty assessment	PICP of PI90	89.9	83.3	82.4	82.5

ME: mean error, RMSE: root mean squared error, MEC: model efficiency coefficient, PICP of PI90: 90 % prediction interval coverage probability.

#### 3.2.3 Uncertainty assessment

**Fig. 5:**
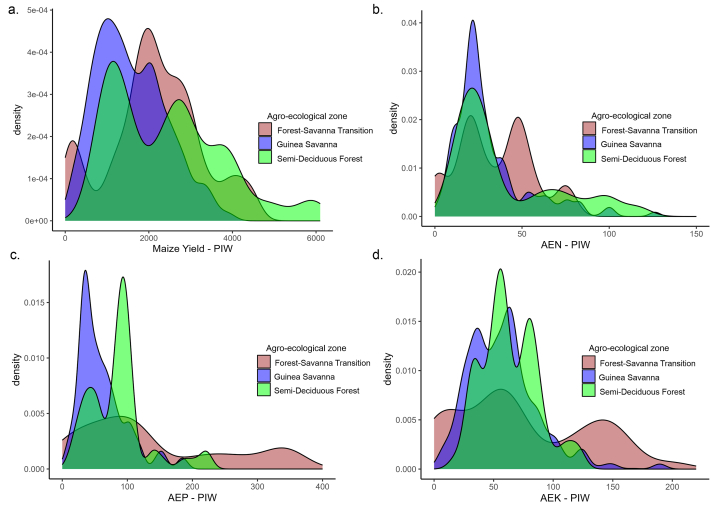
Frequency distribution of PIW90 for a) maize yield prediction across the three major agro-ecological zones, b) AE-N across the three major agro-ecological zones, c) AE-P prediction across the three major agro-ecological zones, d) AE-K prediction across the three major agro-ecological zones.

Fig. 5 shows frequency distributions of the PIW for the predicted yield and agronomic efficiency for the three major maize production agro-ecological zones of Ghana. The figure shows that the PIW distribution for yield is fairly symmetrical while those of the agronomic efficiencies are right-skewed. This indicates that for agronomic efficiencies, the prediction intervals are very wide in some cases, particularly for the FST and SDF zones. The PIW distributions of yield are also fairly wide, in particular for the FST and SDF zones (Fig. 5a), indicating that there are large differences in prediction uncertainty between sites in each zone. The mean and median of the PIW for yield for GS are smaller than those for FST, which implies that for GS the PIW is generally smaller. This indicates that yield predictions in GS tend to be more accurate than for FST (Fig. 5a). Fig. 5b, c, and d indicate that the PIW distributions of AE-N, AE-P, and AE-K are widest and right-skewed for the FST zone, indicating that AE predictions in the FST zone are less accurate than in other zones. The distribution of AE-N within the SDF zone shows a larger mass towards zero than for AE-P and AE-K. This indicates that in this zone the AE-N predictions are more accurate than the AE-P and AE-K predictions. Fig. 5c shows that AE-P predictions have the lowest uncertainty in the GS zone and the highest uncertainty in the FST zone.

**Fig. 6:**
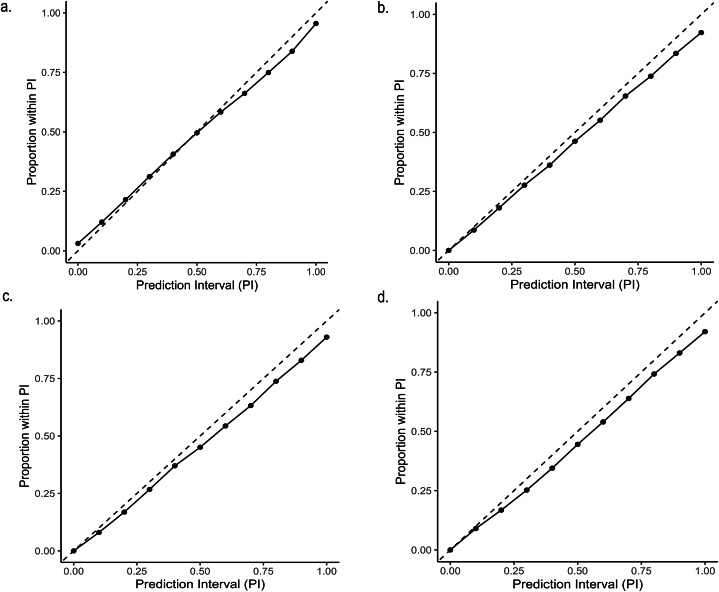
Accuracy plots for PICP of all measurements for a) maize yield, b) agronomic efficiency of nitrogen, c) agronomic efficiency of phosphorus, and d) agronomic efficiency of potassium.

The PICP of PI90 measures the proportion of test values that fall within the 90 % prediction interval. The PICP of PI90 for the yield model was 89.9 %, indicating that the prediction uncertainties were realistically quantified. The PICP of PI90 for the agronomic efficiency of N, P, and K models ranged from 82.4 % to 83.3 %, indicating that the models somewhat underestimated the uncertainties (Table 8). Fig. 6b – d shows that the prediction uncertainty for AE-N, AE-P, and AE-K was underestimated for all PIs. For yield the PICP values were much closer to the 1:1 line, although PIs lower than 0.30 slightly overestimated the prediction uncertainty and PIs above 0.60 slightly underestimated the prediction uncertainty (Fig. 6a).

#### 3.2.4 Relative importance of predictor variables for maize yield and agronomic efficiency predictions

**Fig. 7:**
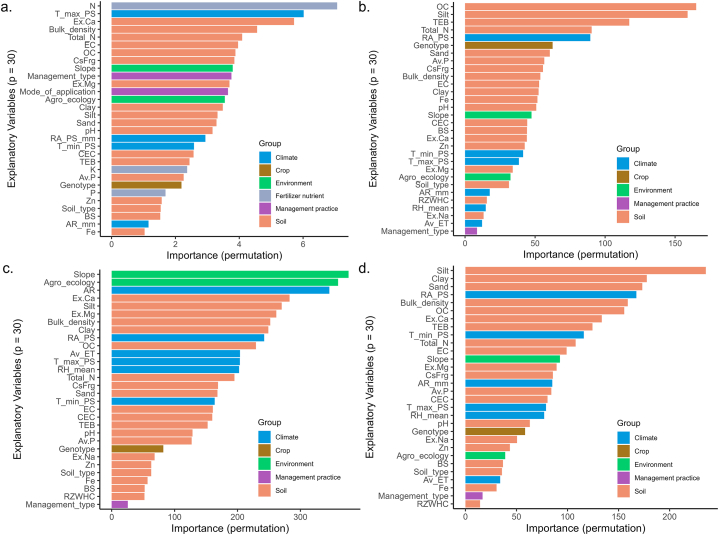
Variable importance plot from RF algorithm determined by the permutation method for: a) maize yield prediction using 3136 data points, b) AE–N prediction using 2145 data points, c) AE–P prediction using 1897 data points, and d) AE–K prediction using 1799 data points.

The variable importance plot (Fig. 7) shows the influence of fertilizer nutrients, soil properties, climatic and environmental variables, crop parameters, and management practices on yield and agronomic efficiency predictions. Fig. 7a shows that maize yield is primarily influenced by the amount of nitrogen fertilizer applied, maximum temperature during the planting season, and exchangeable calcium content of the soil. Bulk density, total nitrogen content, electrical conductivity, and soil organic carbon content follow in importance, indicating that soil is an important predictor variable with 5 out of 7 most important variables. The slope of the terrain, management type, and mode of fertilizer application are also identified as key variables for predicting maize yield. Fig. 7b - d reveal that soil organic carbon, soil texture (with silt being the most influential, followed by clay and sand), the amount of rainfall received during the planting season, and bulk density are important predictor variables for all three agronomic efficiencies. However, there are also notable differences. Slope and agro-ecological conditions are the most important variables for AE–P, while they rank much lower for AE–N and AE–K. A similar observation can be made for total annual rainfall, which is highly important for AE–P but less so for AE–N and AE–K. The variable importance plots show that soil properties contribute the most to yield and agronomic efficiency, followed by climate, crop, and environmental conditions.

**Fig. 8:**
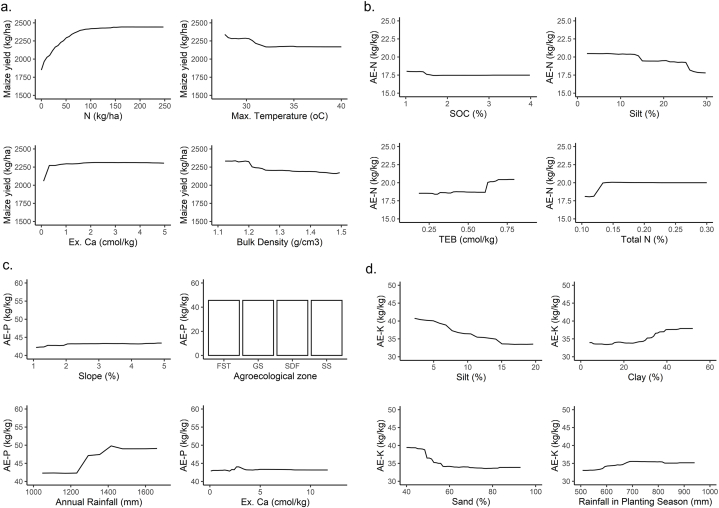
Partial dependence plot of a) maize yield, b) AE-N, c) AE-P, and d) AE-K for the top 4 ranked predictor variables from the variable importance of the RF algorithm. The x-axis plots the range of the predictor variables from the 5 to the 95 percentiles.

The PDPs for yield, AE-N, AE-P, and AE-K are shown in Fig. 8a, b, c and d, respectively. Not surprisingly, nitrogen fertilizer has a positive relationship with maize yield, which increases from 1800 to 2400 kg ha^−1^ as the rate of nitrogen fertilizer increases from 0 to 90 kg ha^−1^ across all agro-ecological zones (Fig. 8a). Increasing the nitrogen application rate even further does not lead to a higher model predicted yield as the PDP curve levels of at nitrogen application rate of 90 kg ha^−1^. An increase in maximum temperature above 30 °C leads to a decrease in the yield, as can be seen in the negative relationship between yield and maximum temperature (Fig. 8a). Fig. 8a shows that there is no significant relationship between exchangeable calcium and maize yield, except for small values of exchangeable calcium, which leads to lower yields. The relation between bulk density and yield is also negative, which could be due to soils rich in organic matter and nutrients tending to have lower bulk density. Fig. 8b shows that soil organic content (SOC) above 1.5 % has no significant effect on AEN. Silt has a marginal negative effect on AE-N, because- AE-N starts to decrease when the silt content of the soil increases from 10 to 30 %. The PDPs of the RF algorithms for AE-P and AE-K show a positive relationship between these AEs and rainfall (Fig. 8c and d). AE-P is constant across all agro-ecological zones even though it ranked second in variable importance. Calcium has no significant effect on AE-P (Fig. 8c) whilst increase in silt content leads to decrease in AE-K (Fig. 8d).

## 4 Discussion

### 4.1 Evaluation of RF algorithm performance and uncertainty assessment for crop production

Nested cross-validation is advantageous in model evaluation as it mitigates the risk of overfitting and provides a more unbiased estimate of model performance [52]. By using an outer loop to split the data into training and test sets, and an inner loop for hyperparameter tuning and model selection, it ensures that the test set remains completely independent of the model evaluation process [52]. This separation is crucial for obtaining realistic performance metrics, as it simulates the real-world scenario where the model encounters unseen data. The robustness of this method lies in its ability to repeatedly test the model on multiple different splits of the data, thus giving a comprehensive view of how the model is likely to perform in practice. The results from the nested cross-validation of this study provided a robust model evaluation approach and demonstrated that the RF algorithm was effective in predicting yield with a MEC of 0.81 and RMSE of 582 kg ha^−1^, which is akin to other studies that used RF for crop yield prediction. The RF algorithm's effectiveness can be attributed to its ability to handle large datasets with high-dimensional features which makes it particularly suited for agricultural data, which often include a multitude of variables such as soil properties, weather conditions, and management practices [29]. For example [29], obtained a MEC of 0.78 and RMSE of 835 kg ha^−1^ when modelling maize yield in Brazil, which indicates that our RF model performed slightly better. This could be due to the larger number of predictor variables included in our study. Similarly [53], found that including more predictor variables in RF predictions improved the accuracy of the model. While the yield model developed in this study performed well, prediction performance for agronomic efficiency of N, P, and K prediction was lower, with MECs ranging from 0.54 to 0.63. Apparently, the predictor variables did not explain the spatial variation of agronomic efficiencies well. This may be due to the fact that in many cases, the response of the crop to fertilizer application was not strong. This observation corroborates with that of [54], who also observed that in plots where soil fertility was high, applying more fertilizer did not have a significant effect on yield. We accounted for this by including soil nutrient concentrations as predictor variables in the RF model, but it remained challenging to predict AE from the predictor variables. Nonetheless, all AE models explained more than half of the AE variance and are therefore considered useful, despite the significant prediction uncertainty.

We did not include fertilizer application as a predictor variable in modelling AE because it would be awkward to include a predictor variable that is already part of the definition of the AE (Eq. (1)). For example, if we used N application as a predictor variable, it would make more sense to predict yield gain using a RF model and then divide the result by the known N application to obtain a prediction of AE-N, rather than predicting AE-N directly from a model that includes N application and other predictor variables. This would allow us to better utilize the known N application. While this approach could potentially improve model performance, it was outside the scope of this research. Including fertilizer application as a predictor variable would likely have a high impact on AE predictions and diminish the effect of other predictor variables, whereas this study focused mainly on the influence of these predictor variables on AE. Therefore, we recommend that future research compare machine learning prediction of AE with and without including fertilizer application as a predictor variable. It is important to note that including fertilizer application as a predictor variable means that AE predictions are dependent on the fertilizer application rate, resulting in AE prediction that are not constant but vary with N, P, and K application rates.

The optimized hyperparameter used to predict yield resulted in an RF algorithm that explained 81 % of the variation in the data (Table 8). However, despite the optimized hyperparameters being the same for the agronomic efficiencies, the models explained different amounts of variation, ranging from 54 to 63 %. A study by Schratz et al. [55] reported no significant effect of hyperparameter tuning in RF modelling and concluded that the RF algorithm often produces accurate results with default hyperparameter values. We observed that the default hyperparameters for the RF algorithm in our study performed similarly to models with optimized hyperparameters (results not shown). This suggests that, in this study hyperparameter tuning was not a crucial step in RF modelling.

The PIW and PICP results obtained using the RF algorithm for yield prediction showed that the prediction uncertainty was realistically quantified. However, the assessment of uncertainty for agronomic efficiencies showed greater deviations from the ideal value, indicating that the models were less reliable in quantifying uncertainties compared to yield prediction. This could be attributed to the fact that the models for agronomic efficiencies were trained on a skewed dataset that had many extreme values (Table 3). Additionally, we observed that the PIW assessment for the agronomic efficiencies in the GS was narrower compared to the FST and SDF zones. This observation may be explained by the model performing more accurately within a zone that had a greater number of trial data, for example, in the case of the GS zone (Fig. 1; Table SI 12) and a more even distribution within the zone. On the other hand, the FST and SDF agro-ecological zones had fewer field trials data and a less uniform distribution across the zones (Fig. 1; Table SI 12). These zones also exhibited less local spatial distribution, making accurate predictions more challenging. Our findings support those of [56], who reported that uncertainties in the model's predictions were predominantly large in areas with substantial spatial variability and limited data points to capture the spatial variations. Areas with high uncertainty predictions can lead to risk-aversion behavior among farmers or stakeholders, potentially limiting the adoption of innovative practices. This can result in suboptimal resource allocation leading to lower productivity. For example, if a model predicts crop yield with high uncertainty in a certain zone, farmers may be reluctant to invest in inputs such as fertilizers or high-quality seeds, etc., due to concerns about returns on investment. Farmers can make better informed decisions based on such models' results to avoid incurring significant losses. To improve model predictions in such zones, the limited data available should be improved with more data for model calibration.

### 4.2 Implications of variable importances for yield and agronomic efficiency for sustainable agriculture

Fig. 7 showed the importance of soil exchangeable calcium in driving maize yields and agronomic efficiency of N, P, and K, as this parameter ranked high in determining all four dependent variables, possibly due to the crucial role it plays in stabilizing soil aggregates and in improving soil structure [57] to enhance nutrient availability for plant uptake. Our findings corroborate a review by Zingore et al. [54] which identified exchangeable calcium as one of the important determinants of maize yields in SSA. In a study by Mtangadura et al. [58], the authors identified that a decline in maize yields was linked to the depletion of soil exchangeable Ca, Mg, and K. The deficiency of calcium in the soils of Ghana, as a result of nutrient leaching, leads to decreased pH levels [59]. found that applying 2.5 t ha^−1^ lime to acidic soils in the GS agro-ecological zone of Ghana improved soil fertility and increased yield coupled with improved efficiency of fertilizer applied. Our study also revealed that rainfall during the planting season plays a significant role in maize yield and agronomic efficiency [60]. Since most cropping systems in SSA are rainfed, the inclusion of supplementary irrigation could be beneficial, especially in the context of climate change [61].

The role of soil texture in influencing maize yields and agronomic efficiencies of N, P, and K was evident in our results, supporting the findings of Kihara and Njorege [60] who observed increased phosphorus agronomic efficiency as a result of higher soil silt content. Soil texture, due to its impact on the physical and chemical properties of the soil viz. water-holding capacity, aeration, nutrient availability, and root growth, is an important consideration in crop production. The dominant soil types (e.g. Lixisols) in the GS agro-ecological zone (Figure SI 4), generally have sandy to sandy loam textures, which are susceptible to nutrient leaching due to low soil organic carbon content [62]. Consequently, our results also clearly indicate the role of soil organic carbon in yield and agronomic efficiency [63]. In this study nitrogen fertilizer application emerged as the most important determinant of yield due to its crucial role in plant growth. Our findings corroborate with those of [64], who identified nitrogen as the most yield-limiting nutrient, and [65], who found that nitrogen application accounted for the largest yield response in maize production in SSA. This emphasizes the need for effective nitrogen management in cropping systems in SSA to enhance crop productivity for sustainable agriculture [1,66,67].

The agronomic efficiency of nitrogen was mainly influenced by soil organic carbon, confirming the findings of [68,69], who call for remedial measures of soil organic matter management in cropping systems. Our analysis suggests that adequate increase in soil organic carbon content will improve agronomic efficiencies. As an indicator of soil fertility, organic carbon plays an essential role in nitrogen agronomic efficiency [70]. Furthermore, carbon and nitrogen are stoichiometrically linked in the soil matrix. Thus, an increase in soil carbon indicates an increase in nitrogen concentration [71].

The RF algorithm identified soil texture as an important variable for the agronomic efficiency of potassium, confirming an earlier study by Rosolem and Steiner [72] who reported that in tropical soils, soil clay content plays a significant role in the movement of potassium fertilizer within the soil profile. In the context of Ghanaian soils, soil texture can have significant effects on the leaching of fertilizers [73]. noted that the GS zone of Ghana predominantly has sandy-textured soil with high permeability and low water-holding capacity, leading to high leaching losses of fertilizers and reduction in the effectiveness of fertilizers. Although soil and climatic variables were both important variables for yield prediction, the soil was identified as most important in this study. This may be due to the high soil variation in the Ghanaian landscape compared to climate [73]. Higher variation means a potentially bigger effect on yield because predictor variables that are nearly constant cannot explain spatial variation. Also, most of the maize trials’ datasets did not have weather information for the location but relied on the nearest rainfall station, which lead to the climatic datasets for some experiments being the same. In contrast, most of the trials had their soil information from soil samples analyzed from the field and as such the soil variables varied from experiment to experiment, except in limited instances where some missing data were replaced with soil information from maps.

### 4.3 Partial dependence analysis and implications for food security

The partial dependence analysis was conducted based on the RF algorithm for predicting maize yield and agronomic efficiency with the resulting PDP confirming the importance of nitrogen fertilizer application in maize cultivation. The PDP for yield (Fig. 8a) showed an increase in maize yield to 2400 kg ha^−1^ as nitrogen fertilizer application increased to 90 kg ha^−1^, above which there was no more significant increase in yield. Though other factors may come into play based on local soil conditions, our findings largely confirm earlier results of [10], recommending 90 kg N ha^−1^ as the economic application rate for maize production in Ghana. From the PDP, we observed a decline in maize yield as temperatures exceed 30 °C, possibly due to induction of physiological stress in the maize plant at high temperatures, leading to reduced growth and development. This stress can result in decreased root growth, impaired nutrient uptake, and increased susceptibility to pests and diseases, which negatively impact maize yields. Our findings corroborate those of [74], who found maize vulnerability to heat stress (>30 °C) and reported a strong reduction in yield above this threshold.

Additionally, we observed that rainfall had a positive relationship with agronomic efficiency, as also reported in Vanlauwe et al. [75]. This can be explained from a direct effect of better moisture conditions on improved rooting density, improved nutrient mobility in the rooting zone, and a higher microbial activity releasing additional nutrients from soil organic matter [75]. To maximize the benefits of rainfall for agronomic efficiency, several management practices can be implemented. The application of organic amendments to improve soil structure and nutrient availability, along with mulching and cover cropping to enhance soil moisture retention [76], is essential to optimize fertilizer utilization in maize production [77].

It is important to note that the findings above need to be interpreted with care. Our study was based on observational data and analyzed with a statistical model, which means that relations found are based on correlations and do not necessarily assess causalities [78,79]. For instance, found relations might be the result of hidden, confounding variables. To determine causalities, it would be necessary to conduct properly designed field experiments [78], which is feasible for control variables such as fertilizer application and management, but much more challenging or practically impossible for other variables, such as soil texture, soil organic carbon, rainfall, temperature and evapotranspiration.

### 4.4 Impact of this study

This study applied a RF machine learning approach to predict maize yield and agronomic efficiency in Ghana and identified the most important predictor variables. Our findings suggest that the model holds significant potential for deriving site-specific fertilizer recommendations, thereby enhancing nutrient use efficiency. The results of the PDP of Fig. 8a showed an average effect of N application on yield and suggested that, on average, an application rate of 90 kg N ha^−1^ would be sensible. However, the model allows for deriving this relationship for specific locations with different conditions and values of other predictor variables. This means that for some cases, 90 kg N ha^−1^ is optimal, but for other cases, this might be another rate, such as 75 kg N ha^−1^ or 100 kg N ha^−1^. Indeed, the model can plot the yield response to fertilizer application for each individual case. Thus, it is a tool that can be used for deriving site-specific fertilizer recommendations. Providing site-specific targeted recommendations, reduces the risk of over-fertilization, thus preventing environmental degradation through nutrient leaching and runoff. Moreover, improved fertilizer use efficiency can translate into economic benefits for farmers by lowering input costs while maintaining or even increasing crop yields. This fosters sustainable agricultural practices by promoting responsible resource utilization and mitigating the negative ecological impacts associated with excessive fertilizer application. Furthermore, it would be very interesting for future study to put recommendations derived from the machine learning model to the test in field experiments and compare them with existing fertilizer recommendation approaches. Again, by understanding the relationship between maize yield and agronomic efficiency and various predictor variables, this can support farmers and other stakeholders to make informed decisions to maximize yields and implement management practices towards improving agronomic efficiency. Soil variables were observed to have a substantial influence on agronomic efficiency. Hence, management practices such as application of organic amendments to improve soil condition, moisture retention with mulching and cover cropping should be incorporated into farming practices to improve soil condition for maximum efficiency. Overall, the integration of machine learning in agricultural decision-making facilitates precision agriculture approaches, promoting sustainability in modern farming practices.

### 4.5 Limitations of this study

This study demonstrated that machine learning models can contribute to improving food security in Sub-Saharan Africa by predicting yields and identifying driving factors and agronomic efficiency. This can guide stakeholders in making decisions for sustainable agriculture. However, there are limitations to this study that need to be addressed in future research. For example, the models had limited performance and could not explain all variations in yield and agronomic efficiency. This is likely because the models lacked other important predictor variables, such as agronomic practices, pest and disease infestation, and cropping history information. Unfortunately, these variables were not available in the compiled trial datasets. To address this limitation, research trials managers should report this information, and future research should collect and incorporate these predictor variables to develop more comprehensive and accurate models.

It is important to note that while the Random Forest algorithm has proven to be effective in this study, advanced machine learning models beyond Random Forest could also be applied which may lead to further improvement in prediction. These models including Extreme Gradient Boosting [80], Artificial Neural Networks [81], and Support Vector Machines [82], may also enhance prediction accuracy.

Although this study was based on a fairly large dataset, a larger training dataset would be ideal. Therefore, continued efforts are needed to collect more data covering different seasons to train these models. Additionally, the quality of training data is crucial. There are significant measurement discrepancies in both the dependent and predictor variables. For example, gap filling was used for some field trial data, which affected the quality of these data. Yield data are also prone to measurement errors due to the lack of standardized protocols.

Another limitation of this study was that data-driven machine learning models cannot easily be extrapolated to situations outside the training data. Therefore, the use of the model is restricted to situations covered by the training data [83]. Applying the model for extrapolation is risky and may lead to lower performance, especially when using the model in other parts of the world or even other parts of West Africa.

## 5 Conclusion

This study assessed the performance of the RF machine learning algorithm for predicting maize yield and agronomic efficiency of nitrogen, phosphorus, and potassium in Ghana and assessed the uncertainties associated with the models’ predictions. We conclude that the RF machine learning algorithm can efficiently predict yield and agronomic efficiency of the nutrient using the available predictor variables. Based on the yield prediction model, we showed that nitrogen application beyond 90 kg ha^−1^ does not lead to substantial yield increase across all agro-ecological zones of Ghana. Soil variables were important drivers of yield and agronomic efficiency, hence, management practices including application of organic amendments to improve soil condition should be incorporated into farming practices for maximum efficiency. Overall, this research provided much insight into the driving factors for maize yield and agronomic efficiencies in a tropical climate and can guide development of management and fertilizer nutrient recommendations for sustainable maize production in SSA.

## Funding

This research was funded by the 10.13039/100016566Mohammed VI Polytechnic University, Morocco and the FERARI project.

## Data availability statement

The data will be made available on request.

## Code availability

The code used to produce the results of this research are available in a github repository. https://github.com/AsamoahEric/Modelling-Yield-and-AE-with-RF.git.

## CRediT authorship contribution statement

**Eric Asamoah:** Writing – review & editing, Writing – original draft, Visualization, Validation, Software, Methodology, Formal analysis, Data curation, Conceptualization. **Gerard B.M. Heuvelink:** Writing – review & editing, Validation, Supervision, Project administration, Methodology, Conceptualization. **Ikram Chairi:** Writing – review & editing, Validation, Supervision, Methodology, Conceptualization. **Prem S. Bindraban:** Writing – review & editing, Project administration, Methodology, Funding acquisition, Conceptualization. **Vincent Logah:** Writing – review & editing, Validation, Supervision, Methodology, Conceptualization.

## Declaration of competing interest

The authors declare that they have no known competing financial interests or personal relationships that could have appeared to influence the work reported in this paper.
