# Supplementary material for: Random forest machine learning for maize yield and agronomic efficiency prediction in Ghana
Source: Heliyon. 2024 Aug 28;10(17):e37065. doi: 10.1016/j.heliyon.2024.e37065 (PMC11403005; doi:10.1016/j.heliyon.2024.e37065)
Supplement: Multimedia component 1 [file mmc1.docx]

**Supplementary information**

Table SI 1: General information recorded for each research trial

| Acronym | Description | Source |
| --- | --- | --- |
| Report_ID | Unique reference code for each report/experiment | Authors database |
| Author | The publication/report author | Authors database |
| Gender | Gender of the farmer | Authors database |
| Description_of_experiment | Summary of the experiment | Authors database |
| District | District where the experiment was conducted | Authors database |
| Community | Locality where the experiment was conducted | Authors database |
| Region | Region within which the experiment was conducted | Authors database |
| Latitude | Location of the place north or south of the equator where the experiment was conducted | Authors database |
| Longitude | Location of the place east or west of the prime meridian where the experiment was conducted | Authors database |
| Agro_ecology | Specific agro-ecological zone within which the experiment was conducted | Authors database |
| Type_of_trials | Type of research experiment whether on-station or on-farm | Authors database |
| Year_of_experiment | Year within which the experiment was conducted | Authors database |
| Season | Growing season within which, the experiment was conducted | Authors database |
| Av_Start_Month | Starting month when the experiment was initiated | Authors database |
| Av_End_Month | Month within which the experiment ended | Authors database |
| Av_Nu_Days | The average number of days for the whole experiment cycle from planting to harvesting | Authors database |
| Institution | Institution where data were collected | Authors database |
| Reference | Source of the data/information | Authors database |

Table SI 2: Climatic data

| Acronym | Description | Source |
| --- | --- | --- |
| Report_ID | Unique reference code for each report/experiment | Authors database |
| Site_ID | Unique reference code for a site from each report/experiment | Authors database |
| Monthly_rainfall_mm | Total monthly rainfall recorded during cultivation of crop | https://www.meteo.gov.gh |
| Av_ET | Average evapotranspiration recorded during the planting season measured in millimeters | https://www.meteo.gov.gh |
| T_min_PS_°C | Minimum temperature recorded in degree Celsius during cultivation of crop in growing period | https://www.meteo.gov.gh |
| T_max_PS_°C | Maximum temperatures recorded in degree Celsius during cultivation of crop in growing period | https://www.meteo.gov.gh |
| RH_mean | Average relative humidity recorded during the cultivation of crop in growing period | https://www.meteo.gov.gh |
| RA_PS_mm | Total rainfall recorded during the planting season measured in millimeters | https://www.meteo.gov.gh |
| AR_mm | Total annual rainfall measured in millimeters | https://www.meteo.gov.gh |

Table SI 3: Crop data

| Acronym | Description | Source |
| --- | --- | --- |
| Site_ID | Unique reference code for a site from each report/experiment | Authors database |
| Genotype | Name of crop variety planted | Authors database |
| Grain_yield | Maize dry grain yield harvested on the plot measured in kilogram per hectare | Authors database |

Table SI 4: Soil characteristics and environmental variables information

| Acronym | Description | Source |
| --- | --- | --- |
| Soil_type | Soil name and type on which experiment was conducted | https://www.csirsoilinfo.org |
| pH | pH of the soil | https://www.csirsoilinfo.org |
| SOC | Organic carbon content of the soil measured in percentage (%) | https://www.csirsoilinfo.org |
| Total_N | Total nitrogen content of the soil measured in percentage (%) | https://www.csirsoilinfo.org |
| CEC | Cation exchange capacity of the soil measured in centi mol charge per kilogram of soil | https://www.csirsoilinfo.org |
| TEB | Total exchangeable bases of soil measured in centi mol charge per kilogram of soil | https://www.csirsoilinfo.org |
| BS | Base saturation of soil expressed in centi mol charge per kilogram of soil | https://www.csirsoilinfo.org |
| RZWHC | Root zone water holding capacity of soil | https://www.csirsoilinfo.org |
| Av P | Available Phosphorus content of the soil measured in milligram per kilogram of soil | https://www.csirsoilinfo.org |
| Ex K | Exchangeable Potassium content of the soil measured in centi mol charge per kilogram of soil | https://www.csirsoilinfo.org |
| Ex Ca | Exchangeable Calcium content of the soil measured in centi mol charge per kilogram of soil | https://www.csirsoilinfo.org |
| Ex Mg | Exchangeable Magnesium content of the soil measured in centi mol charge per kilogram of soil | https://www.csirsoilinfo.org |
| Ex Na | Exchangeable Sodium content of the soil measured in centi mol charge per kilogram of soil | https://www.csirsoilinfo.org |
| Sand | Sand content of the soil measured in percentage | https://www.csirsoilinfo.org |
| Clay | Clay content of the soil measured in percentage | https://www.csirsoilinfo.org |
| Silt | Silt content of the soil measured in percentage | https://www.csirsoilinfo.org |
| BD | Bulk density of the soil measured in cubic centimeter | https://www.csirsoilinfo.org |
| NDVI | Normalized Difference Vegetation Index | Savtchenko et al. (2004) |
| Slope | Slope derived from digital elevation model | https://www.csirsoilinfo.org |

Table SI 5: Fertilizer application and agricultural practices variables

| Acronym | Description | Source |
| --- | --- | --- |
| Plot_ID | Unique reference code for a plot for a particular site | Authors database |
| S_kg_ha | Sulphur fertilizer applied per plot in kilograms per hectare | Authors database |
| Zn_kg_ha | Zinc fertilizer applied per plot in kilograms per hectare | Authors database |
| Fe_kg_ha | Iron fertilizer applied per plot in kilograms per hectare | Authors database |
| N_kg_ha | Nitrogen fertilizer applied per plot in kilograms per hectare | Authors database |
| P_kg_ha | Phosphorus fertilizer applied per plot in kilograms per hectare | Authors database |
| K_kg_ha | Potassium fertilizer applied per plot in kilograms per hectare | Authors database |
| Fertilizer_application_method | Method of fertilizer application adopted | Authors database |

Table SI 6: Number of trials in various agro-ecological zones and soil type

| Soil type | Coastal Savanna | Forest-Savanna Transition | Guinea Savanna | Semi-Deciduous Forest | Sudan Savanna | Total |
| --- | --- | --- | --- | --- | --- | --- |
| Acrisols | 6 (0.4%) | 69 (4.9%) | 605 (42.7%) | 738 (52.0%) | 0 (0.0%) | 1418 (100.0%) |
| Cambisols | 5 (100.0%) | 0 (0.0%) | 0 (0.0%) | 0 (0.0%) | 0 (0.0%) | 5 (100.0%) |
| Gleysols | 0 (0.0%) | 30 (100.0%) | 0 (0.0%) | 0 (0.0%) | 0 (0.0%) | 30 (100.0%) |
| Leptosols | 0 (0.0%) | 0 (0.0%) | 12 (100.0%) | 0 (0.0%) | 0 (0.0%) | 12 (100.0%) |
| Lixisols | 0 (0.0%) | 153 (21.9%) | 507 (72.4%) | 24 (3.4%) | 16 (2.3%) | 700 (100.0%) |
| Luvisols | 0 (0.0%) | 171 (71.2%) | 22 (9.2%) | 47 (19.6%) | 0 (0.0%) | 240 (100.0%) |
| Nitosols | 0 (0.0%) | 0 (0.0%) | 0 (0.0%) | 78 (100.0%) | 0 (0.0%) | 78 (100.0%) |
| Planosols | 0 (0.0%) | 0 (0.0%) | 261 (100.0%) | 0 (0.0%) | 0 (0.0%) | 261 (100.0%) |
| Plinthosols | 0 (0.0%) | 78 (21.4%) | 287 (78.6%) | 0 (0.0%) | 0 (0.0%) | 365 (100.0%) |
| Vertisols | 0 (0.0%) | 0 (0.0%) | 27 (100.0%) | 0 (0.0%) | 0 (0.0%) | 27 (100.0%) |
| Total | 11 (0.4%) | 501 (16.0%) | 1721 (54.9%) | 887 (28.3%) | 16 (0.5%) | 3136 (100.0%) |

Table SI 7: Number of trials in various agro-ecological zones and management type

| Agro-ecological zone | Number of trials by Management type | | |
| --- | --- | --- | --- |
|  | Farmer managed | Researcher managed | Total |
| Coastal Savanna | 0 (0.0%) | 11 (100.0%) | 11 (100.0%) |
| Forest-Savanna Transition | 68 (13.6%) | 433 (86.4%) | 501 (100.0%) |
| Guinea Savanna | 933 (54.2%) | 788 (45.8%) | 1721 (100.0%) |
| Semi-Deciduous Forest | 109 (12.3%) | 778 (87.7%) | 887 (100.0%) |
| Sudan Savanna | 0 (0.0%) | 16 (100.0%) | 16 (100.0%) |
| Total | 1110 (35.4%) | 2026 (64.6%) | 3136 (100.0%) |

Table SI 8: Number of trials in various agro-ecological zones with organic amendment

| Agro-ecological zone | Number of trials with organic amendments | | |
| --- | --- | --- | --- |
|  | No | Yes | Total |
| Coastal Savanna | 11 (100.0%) | 0 (0.0%) | 11 (100.0%) |
| Forest-Savanna Transition | 493 (98.4%) | 8 (1.6%) | 501 (100.0%) |
| Guinea Savanna | 1639 (95.2%) | 82 (4.8%) | 1721 (100.0%) |
| Semi-Deciduous Forest | 847 (95.5%) | 40 (4.5%) | 887 (100.0%) |
| Sudan Savanna | 4 (25.0%) | 12 (75.0%) | 16 (100.0%) |
| Total | 2994 (95.5%) | 142 (4.5%) | 3136 (100.0%) |

 Table SI 9: Number of trials and management type

| Management type | Frequency | % |
| --- | --- | --- |
| Farmer managed | 1110 | 35.4 |
| Researcher managed | 2026 | 64.6 |
| Total | 3136 | 100 |

Table SI 10: Number of trials with organic amendment

| Trials with Organic amendment | Frequency | % |
| --- | --- | --- |
| No | 2994 | 95.47 |
| Yes | 142 | 4.53 |
| Total | 3136 | 100 |

Table SI 11: Number of trials on various soil types

| Soil type | Frequency | % |
| --- | --- | --- |
| Acrisols | 1418 | 45.22 |
| Cambisols | 5 | 0.16 |
| Gleysols | 30 | 0.96 |
| Leptosols | 12 | 0.38 |
| Lixisols | 700 | 22.32 |
| Luvisols | 240 | 7.65 |
| Nitosols | 78 | 2.49 |
| Planosols | 261 | 8.32 |
| Plinthosols | 365 | 11.64 |
| Vertisols | 27 | 0.86 |
| Total | 3136 | 100 |

Table SI 12: Number of trials in various agro-ecological zone

| Agro-ecological zone | Number of Trials | |
| --- | --- | --- |
|  | Frequency | % |
| Coastal Savanna | 11 | 0.35 |
| Forest-Savanna Transition | 501 | 15.98 |
| Guinea Savanna | 1721 | 54.88 |
| Semi-Deciduous Forest | 887 | 28.28 |
| Sudan Savanna | 16 | 0.51 |
| Total | 3136 | 100 |


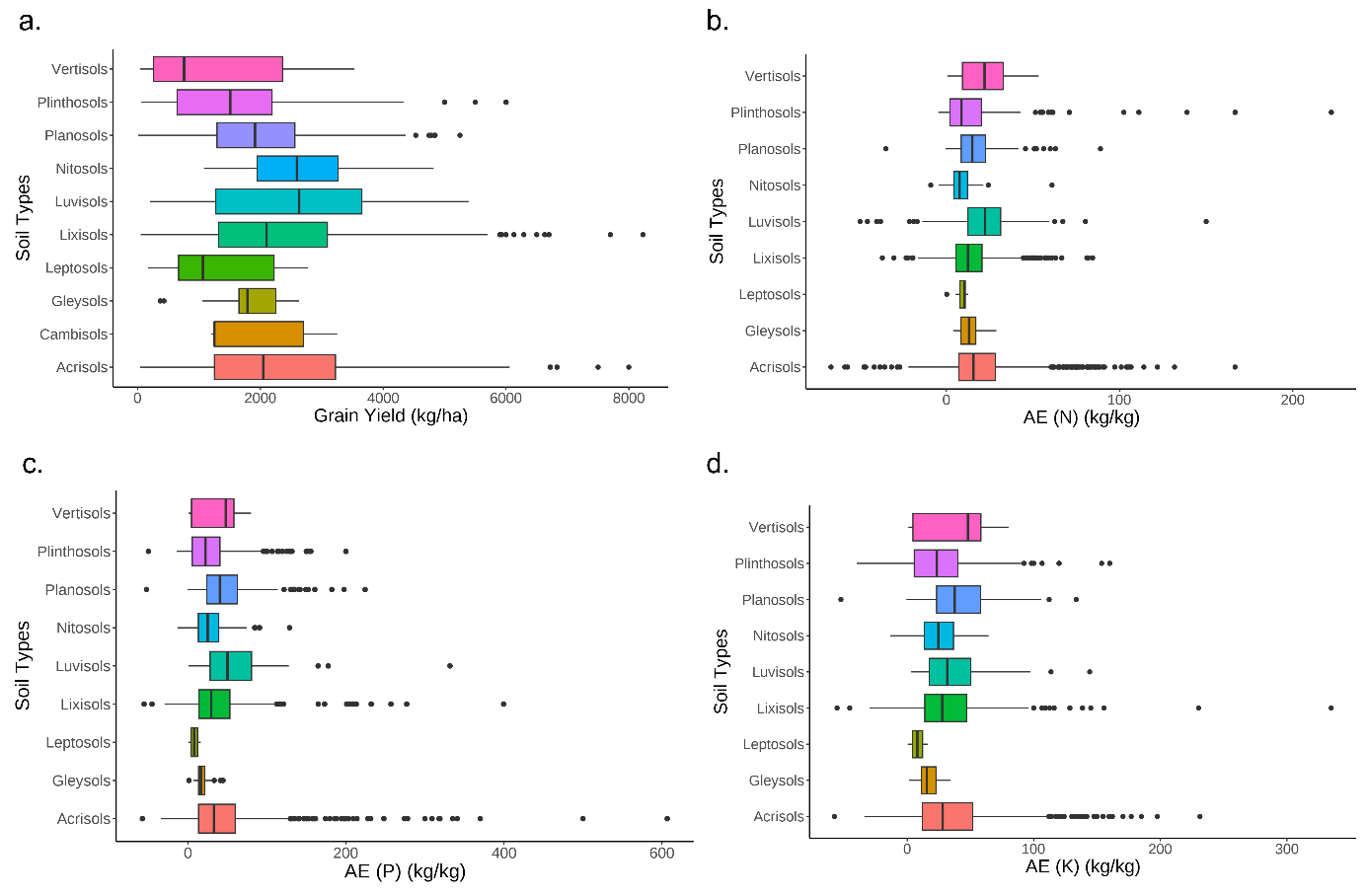


Figure SI 1: Box plots showing the response of a) maize yield, b) AE-N, c) AE-P, and d) AE-K per soil type


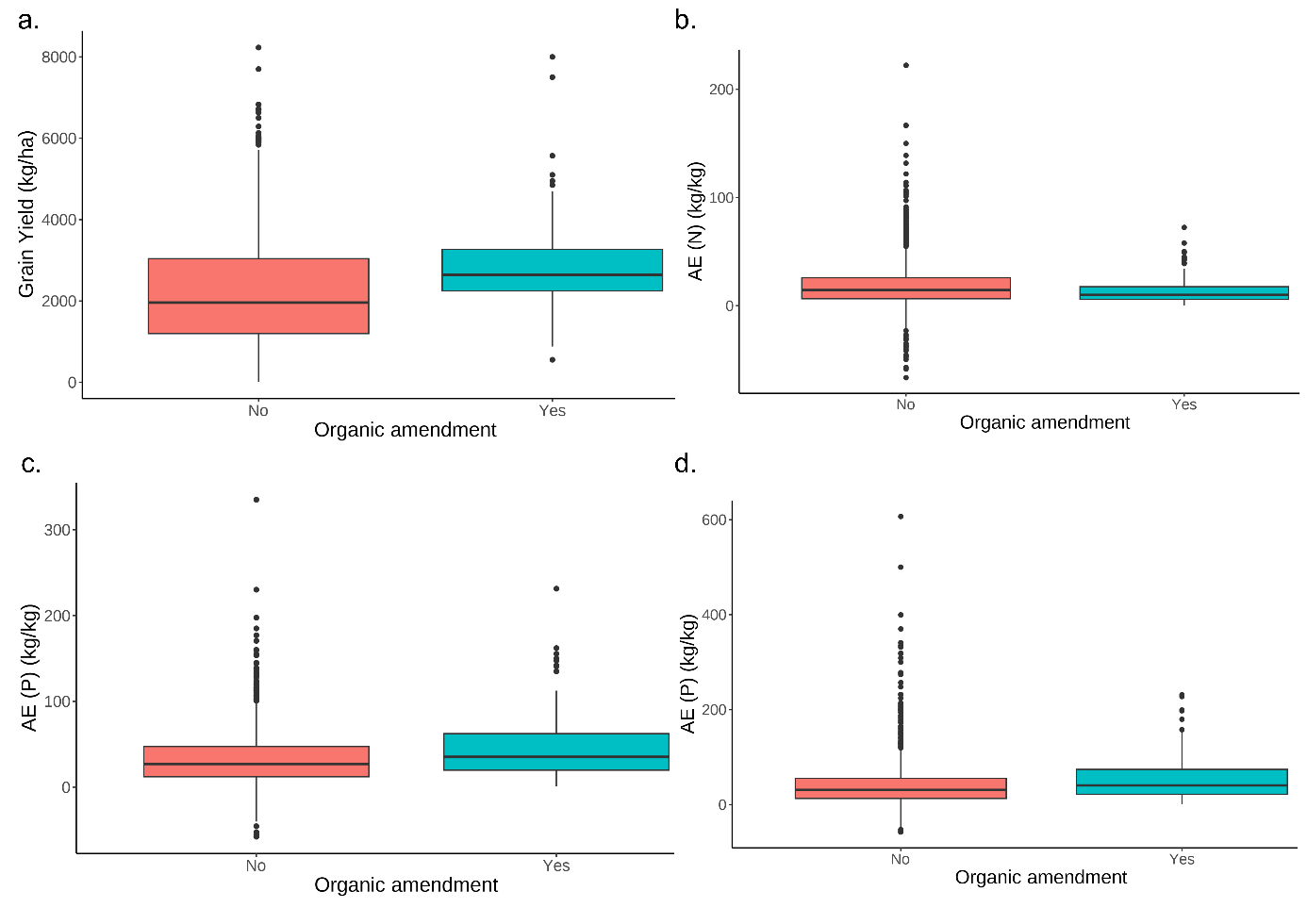


Figure SI 2: Box plots showing the response of a) maize yield, b) AE-N, c) AE-P, and d) AE-K per inclusion of organic amendment


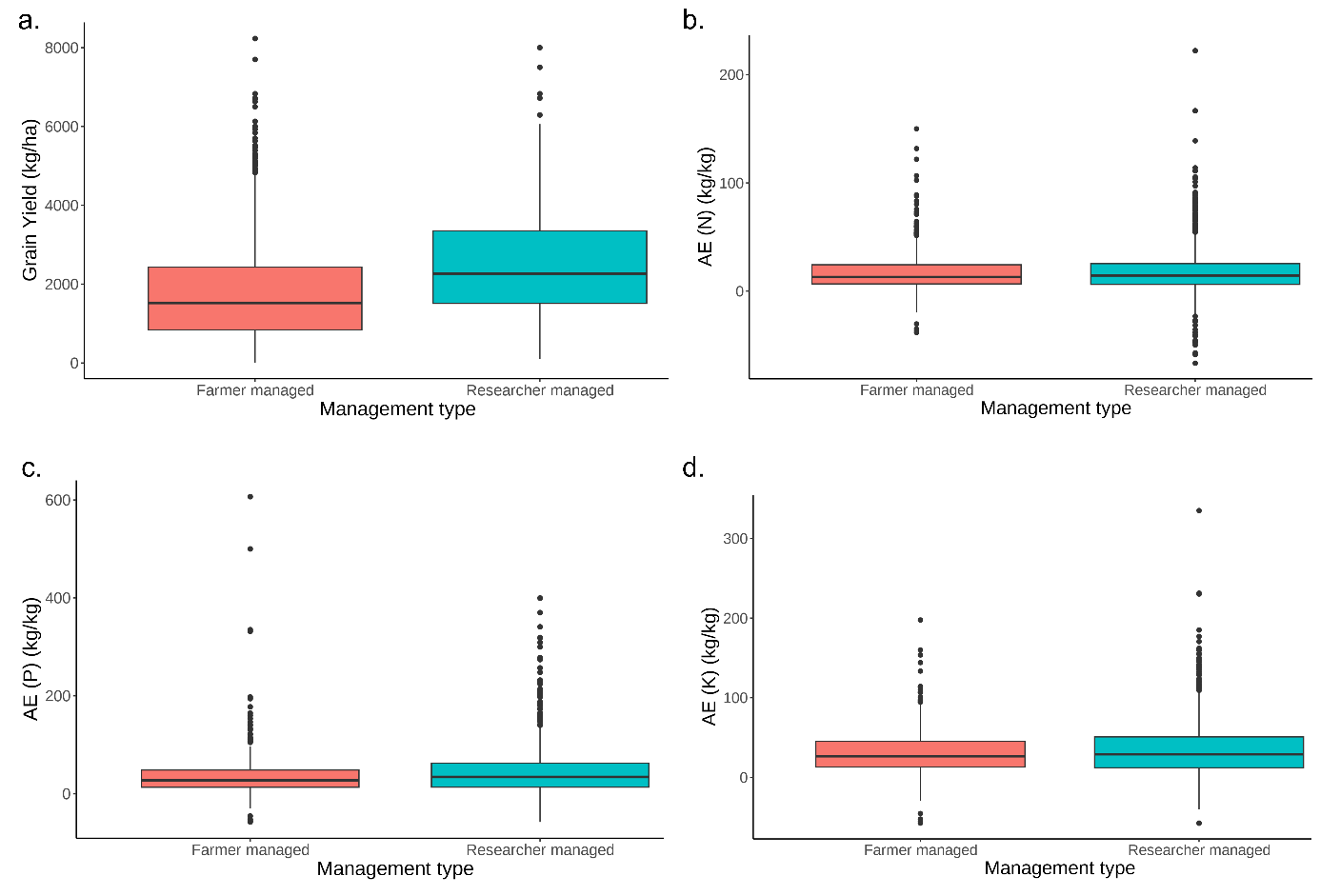


Figure SI 3: Box plots showing the response of a) maize yield, b) AE-N, c) AE-P, and d) AE-K per management type


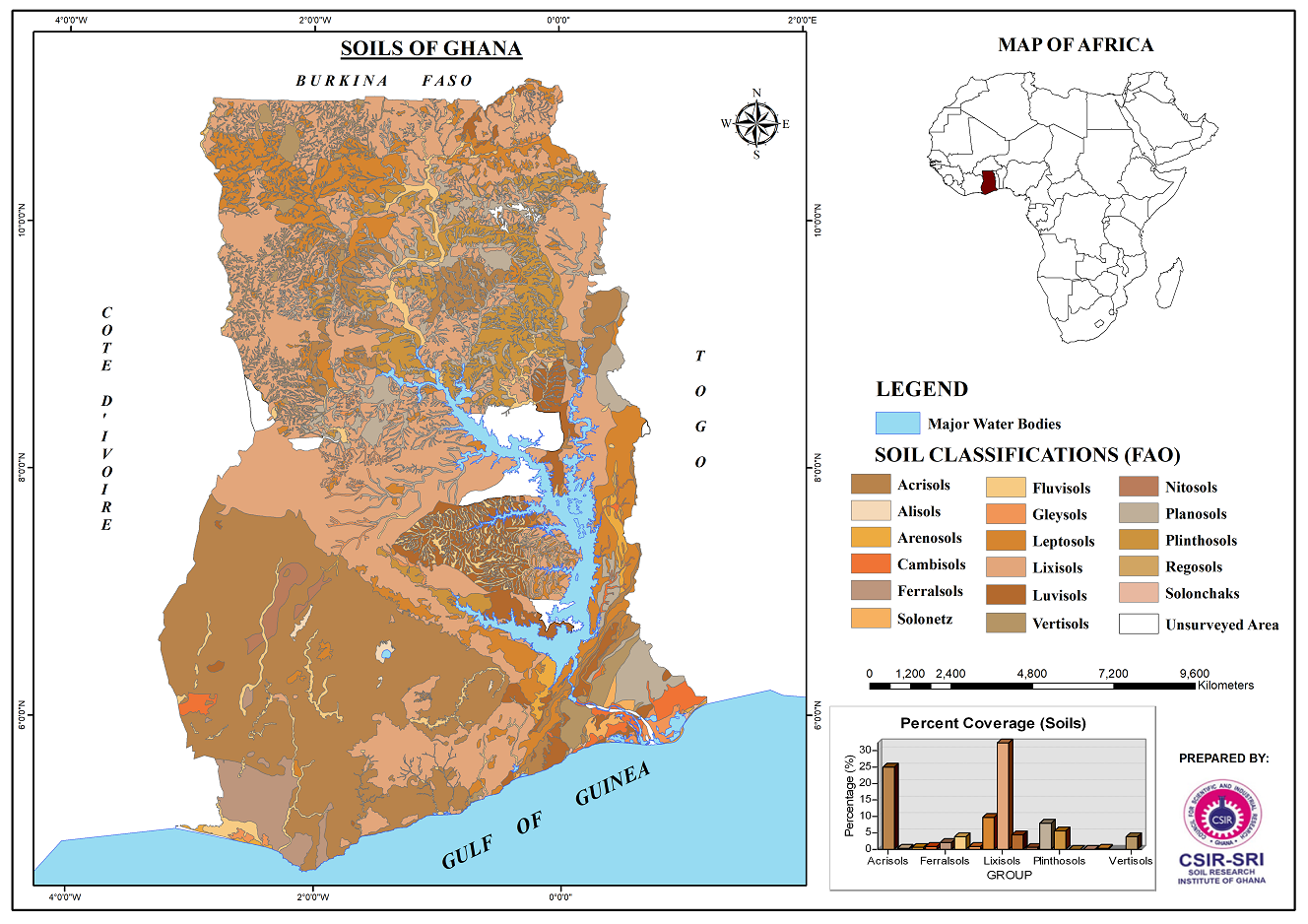


Figure SI 4: Soil map of Ghana
